# Transcriptome provides potential insights into how calcium affects the formation of stone cell in *Pyrus*

**DOI:** 10.1186/s12864-021-08161-5

**Published:** 2021-11-17

**Authors:** Xingyu Tao, Min Liu, Yazhou Yuan, Ruonan Liu, Kaijie Qi, Zhihua Xie, Jianping Bao, Shaoling Zhang, Katsuhiro Shiratake, Shutian Tao

**Affiliations:** 1 State Key Laboratory of Crop Genetics and Germplasm Enhancement, Nanjing Agricultural University, Nanjing, 210095 China; 2 College of Plant Science, Tarim University, Ala’er, China; 3 Laboratory of Horticultural Science, Nagoya University, Nagoya, Japan

**Keywords:** Pears, Stone cell, Lignin, Transcriptome, Calcium

## Abstract

**Background:**

The content of stone cells in pears has a great influence on taste. Stone cells are formed by the accumulation of lignin. The treatment of exogenous calcium can affect the lignin synthesis, but this Ca-mediated mechanism is still unclear. In this study, the author performed a comparative transcriptomic analysis of callus of pears (*Pyrus x bretschneideri*) treated with calcium nitrate Ca (NO_3_)_2_ to investigate the role of calcium in lignin synthesis.

**Results:**

There were 2889 differentially expressed genes (DEGs) detected between the Control and Ca (NO_3_)_2_ treatment in total. Among these 2889 DEGs, not only a large number of genes related to Ca single were found, but also many genes were enriched in secondary metabolic pathway, especially in lignin synthesis. Most of them were up-regulated during the development of callus after Ca (NO_3_)_2_ treatment. In order to further explore how calcium nitrate treatment affects lignin synthesis, the author screened genes associated with transduction of calcium signal in DEGs, and finally found CAM, CML, CDPK, CBL and CIPK. Then the author identified the *PbCML3* in pears and conducted relevant experiments finding the overexpression of *PbCML3* would increase the content of pear stone cells, providing potential insights into how Ca treatment enhances the stone cell in pears.

**Conclusions:**

Our deep analysis reveals the effects of exogenous calcium on calcium signal and lignin biosynthesis pathway. The function of *PbCML3* on stone cells formation was verified in pear.

**Supplementary Information:**

The online version contains supplementary material available at 10.1186/s12864-021-08161-5.

## Highlights

·The content of lignin in pear callus treated with 0.5% calcium nitrate will increase.

·A large number of DEGs enriched in secondary metabolic pathways implies a calcium-lignin regulatory network.

·Overexpression of *PbCML3* in pears increased the content of stone cells in fruits.

## Introduction

Pear is one of the most popular fruits in the world. Stone cells, are an important factor influencing the mouthfeel of the pear fruit, comes from pear parenchyma cells which undergo programmed cell death, thickening of secondary cell walls, and deposition of cellulose and lignin [1]. And lignin deposition is an important process to form stone cells [2]. Therefore, regulation of lignin accumulation is the key to reduce the content of stone cells. Lignification is a unique process of higher plants. Its main function is to provide plant morphological support and improve plant resistance to environmental stress [3]. Similar to most angiosperms, the composition of lignin in pear stone cells is mainly composed of guaiacyl-lignin (G-lignin), with a small amount of syringyl-lignin (S-lignin) and p-hydroxyphenyl lignin (H-lignin) [4]. The branching of phenylpropanol metabolism synthesizes three monoalcohols, and then oxidatively polymerizes them into lignin polymers [5], in which calcium is used to affect stems stiffness and stone cells content in plant [6, 7].

However, how calcium regulates the formation of stone cells is still unclear. Many studies have shown that exogenous calcium treatment has an effect on secondary metabolic synthesis of plants, especially lignin synthesis. The main genes activated after exposure to calcium chloride in pear (*Pyrus spp.* L.) leaves are enriched in secondary metabolic pathways [8]. Exogenous calcium was found to regulate the phenylalanine pathway in peony (*Paeonia lactiflora* P.) inflorescence stems [9], increase stems thickness of roses (*Rosa hybrida* L.) [10], and enhance the stiffness of chrysanthemum (*Chrysanthemum morifolium* R.) stems [7]. In addition, the bending of the gerbera (*Gerbera jamesonii* T.) stem is delayed by exogenous calcium treatment. This may be due to the accumulation of lignin and the coupling of pectin molecules, resulting in the harder cell wall [11]. Moreover, a significant increase in the mechanical strength of the inflorescence stems was found in P.lactiflora after exogenous calcium treatment, which might be caused by changes in cell wall composition [12]. In addition, the intracellular imbalance of Ca^2+^ triggers the development of hard end disorder in pear (*Pyrus pyrifolia cv. ‘Whangkeumbae’*) [13]. The lignification process is considered to be affected by calcium, however, the specific mechanism of the effect of exogenous calcium on lignin synthesis is still unclear.

Calcium is mainly found in cell walls and is a basic signal molecule. It plays an important role in regulating plant growth and development [14]. As an essential nutrient, Ca also plays a prominent role in maintaining the structure and function of the cell wall [15]. In order to maintain cell wall integrity and promote plant growth and development, exogenous Ca treatment is usually used to regulate gene expression [16, 17]. In addition, this positive effect of Ca may be related to calcium ion (Ca^2+^) sensors [18]. Ca^2+^ is a ubiquitous second messenger, involved in a variety of cellular processes. The intracellular free Ca^2+^ concentration will change after plants perceive environmental changes. The changes of calcium ions are different for stimulation of different nature, intensity and duration, and the whole biological reaction process requires the decoding and relay of Ca^2+^ sensors [19–21]. Calcium-binding proteins, is a receptor for Ca^2+^ signaling, which is mainly divided into three categories: (1) calmodulin protein (CaM) and calmodulin-like protein (CML); (2) calmodulin phosphatase B protein (CBL); (3) calmodulin-dependent protein kinases (CDPK) [22].

CaM is the most widely distributed and most important calcium-binding protein. The physiological function of CaM has been extensively studied. It participates in the process of photosynthesis, regulates enzyme activity, cell division and differentiation, cell movement and gene expression [23].. As calmodulin-like protein, CML is also the main sensor of Ca^2+^, which regulates various cell functions by regulating the activity of different target proteins. At present, family members of 50 CMLs and 32 CMLs were identified respectively in model plants arabidopsis and rice [24, 25]. Current studies show that CML plays an important role in plant growth and resistance through multiple interactions, such as regulating the morphology and division of plant cells [26, 27], promoting the plant innate immunity through flagellin-dependent signaling pathways [28], responsing to salt, drought and low-temperature stress, etc. [29]. In Rosaceae, CMLs are widely expressed in apples, including roots, stems, leaves, flowers and fruits, and are spatially specific [30]; however, there are few reports of CML in pears.

Here, we performed exogenous calcium treatment and transcriptome sequencing on pear callus, and analyzed the differences in gene expression levels after treatment. Based on the up-regulated of a large number of transcription factors, the hypothesis that exogenous calcium affects the lignin biosynthesis by regulating transcription factors is further confirmed [9]. In addition, we report a novel *PbCML3* gene that plays a central role in lignin accumulation and stone cells formation. However, the specific regulation mechanism of CML on lignin systhesis needs further study.

## Materials and methods

### Plant materials and treatment

Trees of thirty-year-old *Pyrus bretschneideri* ‘Dangshansuli’ (white pear group), grown on *P. betulaefolia* rootstocks in an orchard in Baoying County, Jiangsu Province, China, were used in this study. Fruit samples were collected from the same trees, 10 days after full bloom (DAF). The collected pulp was cultivated for callus.

Tobacco (*Nicotiana benthamiana* L.) which were planted in potting medium or solid agar media and were grown in a room with constant temperature (25 °C) under a 16-h light/8-h dark cycle and a relative humidity of 40–60%.

### Callus culture

Young fruits with good quality, no mechanical damage and the same size after 10 days of flowering were rinsed repeatedly with tap water to clean the surface and remove impurities, and then rinsed several times with distilled water. Green fruits were immersed in anhydrous ethanol (75%) for 30s in the bechtop, then soaked in sodium hypochlorite (9%) solution for 10 min for disinfection. All petri dishes were sterilized in distilled water with high-temperature sterilization for 3–5 times. After being peeled with a scalpel, and the pulp far from the core was cut into thin and even pieces and inoculated into the induction medium for induction culture.

The callus culture medium formulation has been improved and optimized, namely: MS 4.74 g /L+ 1.5 mg /L 2, 4-d + 0.5 mg /L NAA+ 0.2 mg /L TDZ+ 7 g /L AGAR + 30 g /L sucrose + 10 g /L sorbitol + 0.1 mg /L VC, and pH was 5.8. The culture medium was bottled and sterilized at 121 °C for 20 min. The callus subculture was carried out on the ultra-clean working table. The culture temperature should not exceed 25 °C, protecting from light.

### Calcium treatment of callus

Basing on tissue differentiation MS medium, different concentrations of exogenous calcium nitrate were added: 0, 0.1, 0.5%. Callus with good growth condition and consistent growth were selected and inoculated in the new medium. Cut the good callus into uniform callus with a thickness of about 0.1 cm and lay it on the medium to make it fully contact with exogenous calcium. Calcium treatment was carried out on the ultra-clean working table, and the temperature of callus culture should not exceed 25 °C. The culture cycle was 15 days, and samples were taken every 5 days. The culture medium attached to the callus was washed with distilled water, frozen with liquid nitrogen, and stored in a refrigerator at − 80 °C for testing. Callus without treatment was used as control (CK). Observe the callus before each sampling and photograph callus with obvious changes in growth characteristics. Callus growth state, change of apparent color and degree of looseness were recorded. Compare and statistically analyze the effect of different treatments on callus growth state.

### Determination of lignin content

A suitable amount of dried samples was put into the grinding machine for grinding. After that, 0.01 g of the sample was accurately weighed and added about 1 ml of absolute ethanol to the mortar, then grinding it thoroughly into a homogenate. Then, the samples were transferred to a 2 ml centrifuge tube, and fixed with anhydrous ethanol. After centrifugation at 12000 r for 2 min, discard the supernatant, add 2 ml of absolute ethanol and repeat the above steps. After repeating three times, add 1:2 volume ratio of alcohol and n-hexane to wash the precipitate and repeat three times. The centrifuged samples were air-dried at room temperature. The dried sample was transferred to a new 10 ml centrifuge tube, and 2 ml of acetyl bromide/acetic acid solution with a concentration of 25% was added. The sealing film was sealed and placed in a water bath at 70 °C for 30 min, which was inverted every 10 min. 0.9 ml NaOH 2 mol/L was added to terminate the reaction. After adding 5 ml acetic acid and 0.1 ml7.5 mol/L hydroxylamine chloride, the volume was adjusted to 10 ml with acetic acid. 0.1 ml of the reaction solution was brought into a new 2 ml centrifuge tube, diluting with 0.9 ml acetic acid, and the absorbance value of the sample was determined at the wavelength of 280 nm with an enzyme-linked immunoassay. The standard curve of lignin content was prepared with a standard sample of lignin, and the lignin content in each sample was calculated according to the standard curve (the standard products were purchased from Sigma company). The lignin content is expressed as a percentage of the measured value/the measured sample weight. The final data are expressed as mean ± standard deviation.

### Determination of calcium content

Microwave digestion combined with plasma emission spectrometry (ICP) was used to determine the calcium content in callus. Main experimental steps are as follows: soak the sterilizing tube in acid one day and overnight in advance, rinse the sterilizing tube with deionized water several times, put it on the tube rack, and dry it in the oven. The required volumetric flask, funnel and other instruments shall be cleaned by ultrasonic wave for 30 min. After taking them out, they be washed with deionized water and then dried. The dried samples weighed 0.1 g × 3, wrapped with weighing paper and marked. Put the measured sample in a sterilizing tube and add 8 ml deionized water into the outer tube of the sterilizing tube, then add 2 ml nitric acid into the sterilizing tube. Sterilize it in the instrument, cool it to room temperature, then open the sterilizing outer tube. Take out the sterilizing tube and add the sterilizing liquid into the corresponding 50 ml volumetric bottle. Rinse the sterilizing tube with deionized water for 3 times, and finally, use the deionized water to determine the volume. Use 0.45 m filtering membrane of the water system and 5 ml syringe for filtering. Use the filtrate for standby. About 10 ml of the solution with a constant volume was taken and transferred to a 10 ml centrifuge tube for testing. The results were measured by Prodigy inductively coupled plasma emission spectrometer.

Through the course of the callus development, its calcium content and lignin content were measured every 5 days covering nine developmental samples (S1, CK after 5 days; S2, 0.1% Ca (NO_3_)_2_ treatment after 5 days; S3, 0.5% Ca (NO_3_)_2_ treatment after 5 days; S4, CK after 10 days; S5, 0.1% Ca (NO_3_)_2_ treatment after 10 days; S6, 0.5% Ca (NO_3_)_2_ treatment after 10 days; S7, CK after 15 days; S8, 0.1% Ca (NO_3_)_2_ treatment after 15 days; and S9, 0.5% Ca (NO_3_)_2_ treatment after 15 days).

### Transcriptome with RNA-Seq and data analysis

The total RNA extracted from the pyrus callus of the Control and Ca (NO_3_)_2_ treatment at S1 ~ S6 using a MiniBEST Plant RNA Extraction Kit (TaKaRa, Kusatsu, Shiga, Japan) were used for transcriptome sequencing. The Beijing Genomics Institute (Shenzhen, China) used the Illumina HiSeq™ 4000 system (Illumina Inc., San Diego, California, USA) to prepare and sequence 18 libraries (S1 ~ S6, 3 replicates). The sequence data were uploaded to NCBI and generated SRR number (Table S3). In view of the significant difference in the lignin content of callus on the 10th day of culture, S4 ~ S6 were selected for detailed analysis under the same time dimension. After the original reading, Trimmomatic filter was performed by using the short reads assembling program Trinity, whose annotation was performed by using various bioinformatics databases, including nonredundant protein (NR), nonredundant nucleotide (NT), Interpro and gene ontology (GO), Kyoto encyclopedia of genes and genomes (KEGG) [31]. Cufflinks was used to calculate the FPKM after Tophat2 mapping and DESeq2 was used to screen for DEGs (Table S5), our criterion for screening was padj < 0.05 [32]. The functions of DEGs were explored through GO and KEGG pathway analysis, and the q-value ≤0.05 were defined as significant enrichment. This was to identify metabolic pathways that were significantly enriched.

### Gene expression analysis with quantitative real-time PCR (qRT-PCR)

Total RNA of callus and fruit was extracted using the CTAB method, and genomic DNA contamination was removed by using DNase I (Invitrogen). Then 1 μg of RNA was used for the synthesis of the first strand of cDNA using ReverTra Ace-alpha-First Strand cDNA Synthesis Kit (TOYOBO Biotech Co. Ltd.). qRT-PCR analysis and amplification were performed using Light Cycler 480 (Roche) and all reactions were performed according to the Light Cycler 480 SYBR GREEN I Master (Roche) reagent instructions. The relative expression levels were calculated by calculating the average threshold cycle (Ct) for each sample using the 2^-ΔΔCt^ method.

Gene primers were designed and amplified by using Premier 5.0 software (Table S1). In order to further verify the specificity of primers, the designed primers were examined by BLAST. Finally, the α-tubulin (Pbr042345.1) was normalized as reference gene.

### Subcellular localization

Tobacco leaves were transformed according to the method described by Sperschneider et al. (Sperschneider et al., 2017). The LB medium containing 50 mg/L KNA and 100 mg/L RIP was connected to the activated *Agrobacterium* with sterilized lance and cultured in a constant temperature incubator at 28 °C for 36 h. Later, the *Agrobacterium* was collected by centrifugation and resuspended in the infiltration buffer (10 mM MgCl_2_, 10 mM EMS, PH 5.7, 200 mM Acetosyringone) to a final OD600 of 0.8–1.2. Then, the upper leaves were infiltrated after shaking at room temperature (25 °C) for 4 h. After being cultivated for 3–4 days under normal growth conditions, finally, we used a confocal laser scanning microscope (Zeiss LSM 700, Jena, Germany) to examine the infiltrated leaves.

### The function of gene in pome fruit

According to the method from Xue et al. [33], *35 s-pbCML3-gfp* was injected with a needle for the transformation of the pear, and the solid LB medium containing 50 mg/L KNA and 100 mg/L RIP was used to activate the bacteria, which was then cultured in an incubator of constant temperature at 28 °C for 36 h. The monoclonal points on the line of the incubator were selected with the sterilized toothpick, and then the solid LB medium containing 50 mg/L KNA and 100 mg/L RIP was lined for secondary activation and cultured in an incubator at 28 °C for 36 h. The reactivated agrobacterium was scratched into 30 ml LB (KNA + RIP) liquid medium with the sterilized spearhead and cultured in a constant temperature shaker at 28 °C/220 RPM for 12 h. The bacteria solution was poured into a 50 ml centrifuge tube and centrifuged at 5000 RPM for 10 min to collect the bacteria. 10 ml induction medium (it includes 10 mM MgCl_2_, 10 mM MES, 200 mM acetylsyringone and PH is 5.5) was added to each centrifuge tube to resuspend the bacteria, and the bacteria were induced at 60 RPM at room temperature for 4 h on a small shaking table. The OD600 value was adjusted between 0.8–1.2 by fresh induction medium. The fungus liquid was slowly and uniformly injected into the pear of about 35 days with a syringe needle. The injected pear fruit was incubated at 22 °C in the dark for 24 h, and then cultured at 22 °C for 16 h under light for 8 h and then put it in darkness for 7–8 days. The pear was longitudinally cut and dyed, and the lignin content was determined.

### Statistical analysis

All experiments were repeated three times, in a completely random design. The results were analyzed invariance by using the SAS/STAT statistical analysis package (version 6.12, SAS Institute, Cary, NC, USA). The data in the figures means ± SDs and different letters indicated significant differences (*p* < 0.05).

## Result

### Morphological indices and lignin content

**Fig. 1:**
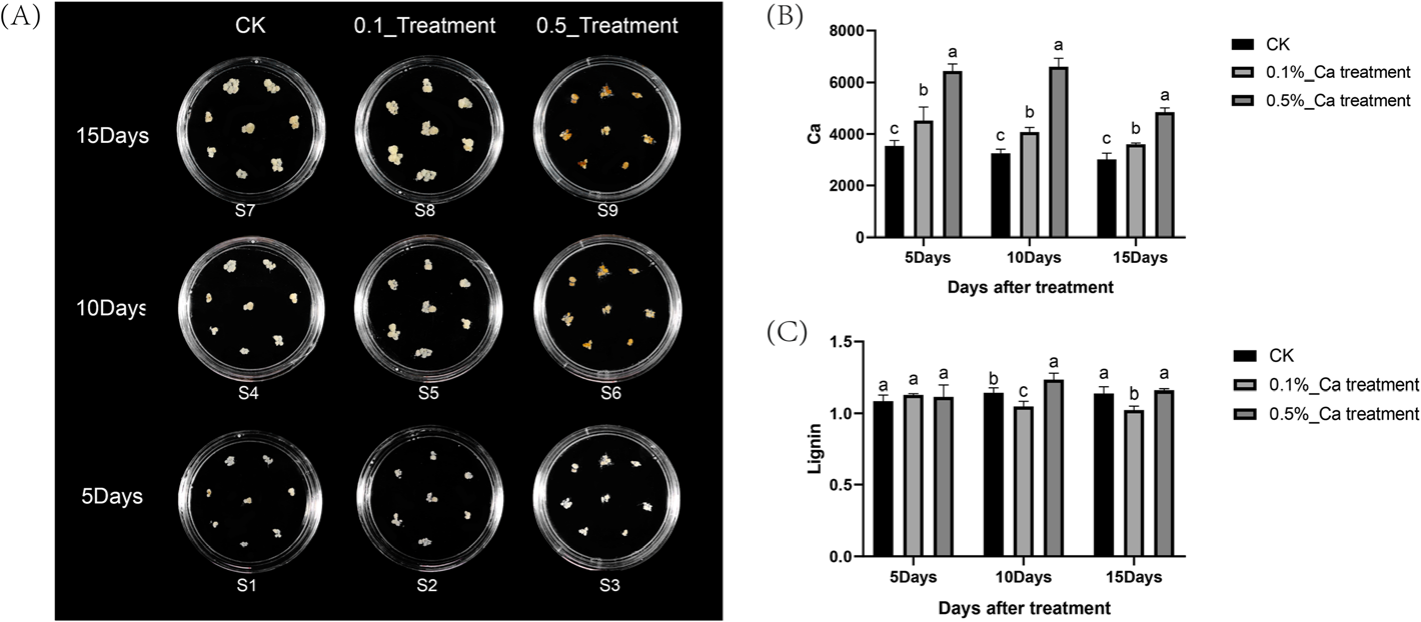
Callus photographs, calcium and lignin content on different treatments. (**A**) Callus treated with different calcium concentrations on 5th, 10th and 15th day. (**B**) Calcium content on different treatments at three stages in callus. (**C**) Lignin content on different treatments at three stages in callus

Callus dealt with 0.5% calcium nitrate showed slight browning comparing to calli treated with CK and 0.1%. The callus treated with CK and calcium nitrate at 0.1% concentration grew faster, and the volume increased rapidly, while callus treated by calcium nitrate at 0.5% grew slower. Callus treated with 0.1% calcium nitrate had a better phenotype than another two treatments on the 10th day, the callus had a loose structure, wet surface, good growth condition and almost no scar. Compared with CK and 0.1% calcium nitrate, the callus treated with 0.5% showed poor growth state, serious browning, and slower volume growth rate (Fig. 1-A).

Compared with the control group, the Ca content in the tissue increased significantly with the treatment of Ca (NO_3_)_2_ in each period (Fig. 1-B). In terms of lignin content, there was no significant difference between the control group and the treatment group in the 5th day. However, there was a significant difference in the 10th day and the 15th day. Compared with S4, the lignin content of S5 decreased while that of S6 increased significantly. Compared with S7, the lignin content of S8 significantly reduced while that of S9 increased slightly (Fig. 1-C). As a result,0.5% Ca (NO_3_)_2_ treatment significantly affects the surface color of callus, and it increased the lignin content of pears’ pulp.

### Transcriptome analysis

**Fig. 2:**
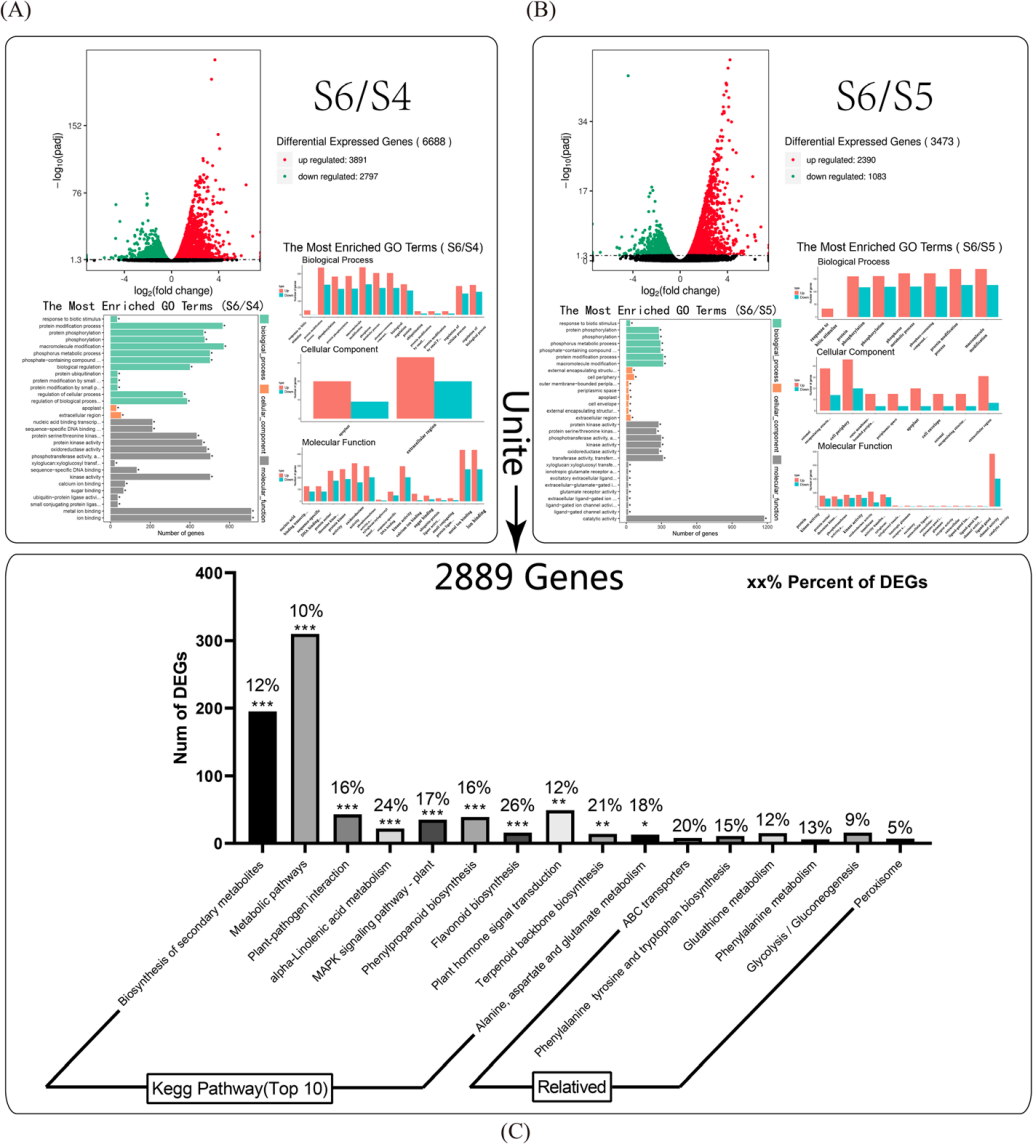
Transcriptome differential analysis. (**A**) Differential analysis of S5 and S4 on the transcriptome, including scatter plots during DEGs screening and GO functional enrichment plots of DEGs. (**B**) Differential analysis of S6 and S4 on the transcriptome, including scatter plots during DEGs screening and GO functional enrichment plots of DEGs. (**C**) KEGG enrichment map based on these intersecting DEGs contains the proportion and number of DEGs in each biological pathways [34–36]

In order to find the changes in the metabolic signals and regulatory network of pear callus after calcium nitrate treatment, three samples on the tenth day were selected for preferable analysis (control [S4], 0.1% Calcium nitrate treatment [S5], 0.5% calcium nitrate treatment [S6]) because of the significant differences with lignin content among them. After comparative analysis between S6 and S4, a total of 6688 DEGs were identified, including 3891 up-regulated and 2797 down-regulated (Fig. 2-A). After comparative analysis between S6 and S5, 3473 DEGs were found, in which the number of up-regulated was 2390, and the down-regulated was 1083 (Fig. 2-B). The intersection of 2889 DEGs between S6/S4 and S6/S5 was obtained.

In order to better classify functional clusters of DEGs, we analyzed the intersection obtained from S6/S4 and S6/S5 by using the online KEGG pathway database (Fig. 2-C). A total of 16 KEGG metabolic pathways were significantly enriched, these metabolic pathways mainly include metabolic pathways, biosynthesis of secondary metabolites, signal transduction of plant hormones, the interaction of plant pathogens, biosynthesis of phenylpropyl, MAPK signaling pathway, metabolism of linolenic acid and biosynthesis of flavonoids. Among them, the metabolic pathways and the biosynthesis of secondary metabolites were participated in the largest number of DEGs, respectively accounting for 10 and 12% of the total number of genes in the two metabolic pathways. In addition, several related metabolic pathways have been identified, including glycolysis, ABC transport, glutathione metabolism, biosynthesis of phenylpropanoid and tryptophan.

Also, three samples on the fifth day were analyzed, 7205 DEGs were obtained by the intersection of S3/S2 and S3/S1. The time dimensions were combined and took the intersection of 5 day and 10 day DEGs to obtain 1473 genes (Table S6). Venn diagrams of DEGs were drawn by R script (Fig. S1).

### DEGs in the shikimate pathway and phenylpropanoid pathway

**Fig. 3:**
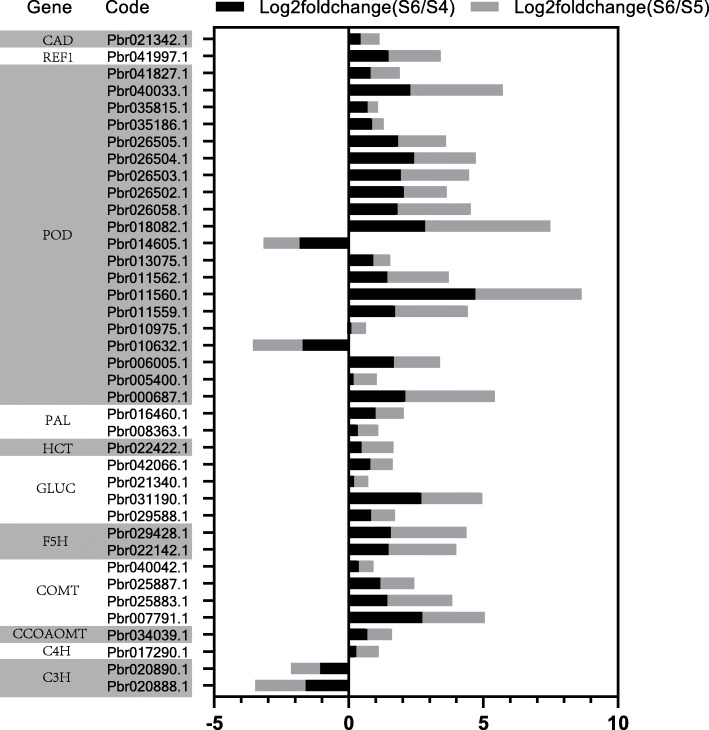
DEGs in phenylpropanoid pathway. The direction and length of the column reflect the differential expression of genes

**Fig. 4:**
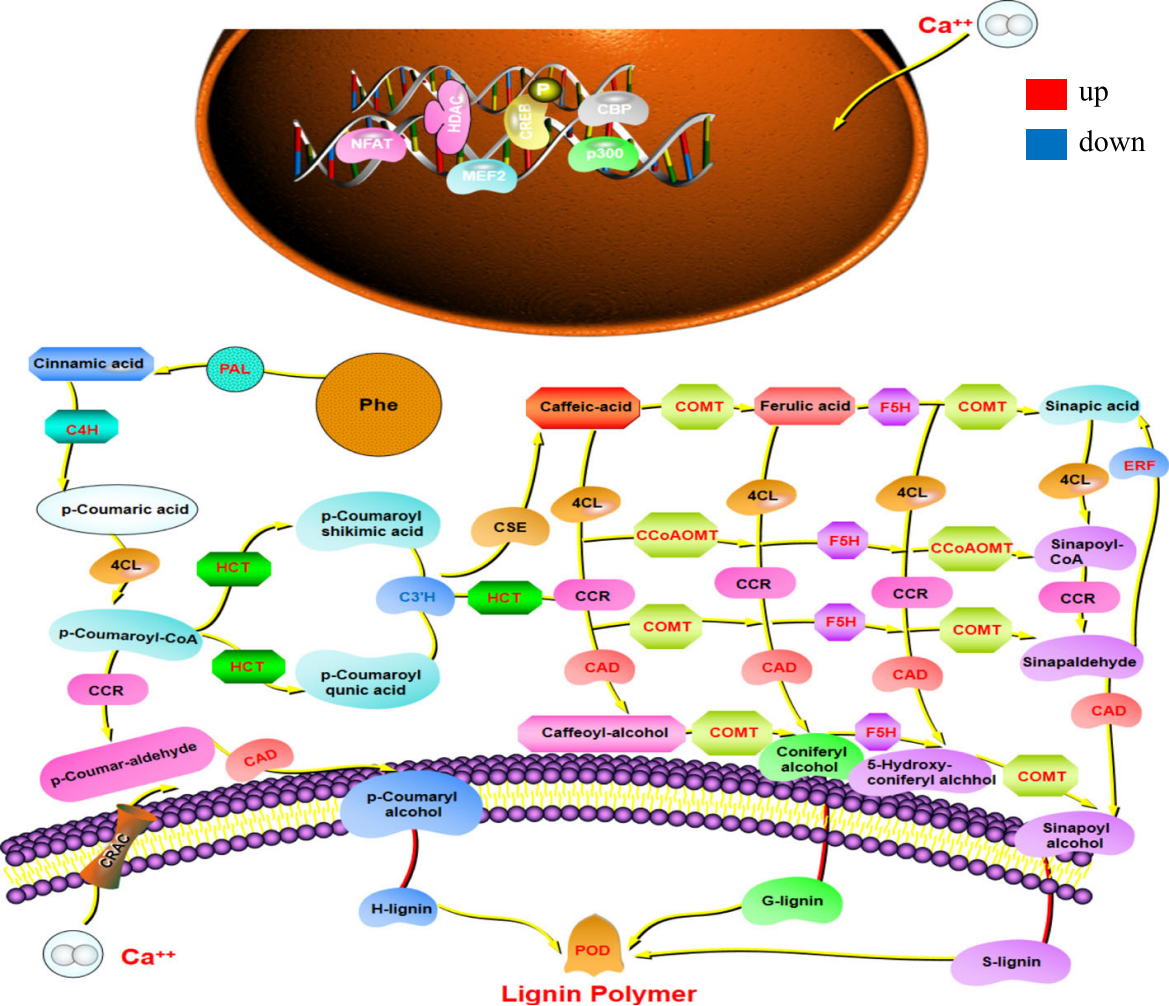
metabolic map of lignin synthesis pathway. Genes with red names indicate up-regulation under Ca (NO_3_)_2_ treatment, and genes with blue names indicate down-regulation under Ca (NO_3_)_2_ treatment

Lignin synthesis is a complex process involving multiple pathways. Related studies have shown that the phenylpropanoid pathway is generally considered to be one of the main pathways for lignin synthesis. PAL, CAD, C4H, C3H and COMT are the key enzymes involved in lignin synthesis in the phenylpropanoid pathway. A total of 11 phenylpropanoid pathway DEGs were selected according to the sequencing results (Fig. 3). Among them, most of the DEGs were up-regulated. Only two C3H encoding genes *Pbr020890.1* and *Pbr020888.1*, and two POD encoding genes *Pbr014605.1* and *Pbr010632.1* showed negative regulation. Among all the DEGs, POD encoding genes *Pbr011560.1* and *Pbr01808.1*, F5H encoding genes *Pbr029428.1* and *Pbr022142.1*, and COMT encoding genes *Pbr007791.1* had obvious and significant differences. Shown as the phenylpropanoid metabolic pathway (Fig. 4), genes with red names indicated up-regulation under Ca (NO_3_)_2_ treatment, and genes with blue names indicated down-regulation. According to the comprehensive analysis, there were some significant changes in phenylpropanoid metabolism pathway, in which the key enzymes involved in lignin synthesis were mainly positively regulated.

**Fig. 5:**
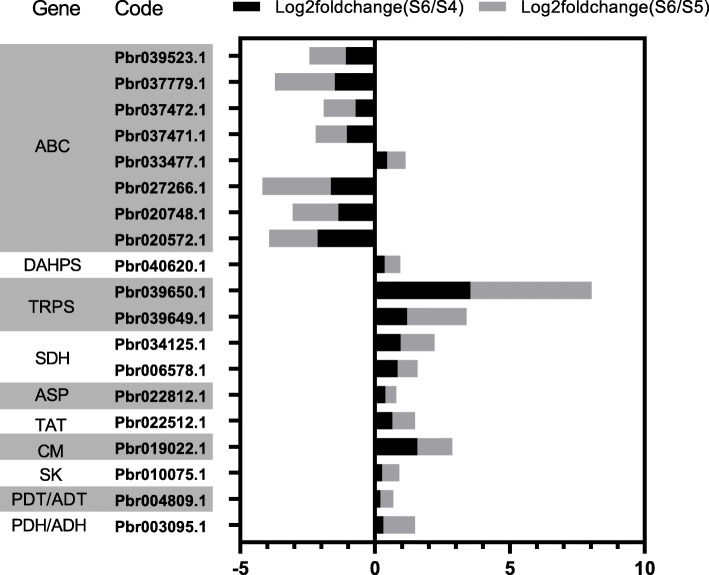
DEGs in shikimate pathways and ABC transporter. The direction and length of the column reflect the differential expression of genes

DAHPS, TRPS, SDH, ASP and ADT are the key enzymes involved in lignin biosynthesis in the shikimate pathway. A total of 9 DEGs were selected according to the sequencing results. Among them, all genes of the shikimate pathway were positive regulation. However, as important transporters in secondary metabolism, ABC transporters are mostly down-regulated (Fig. 5).

### Screening of differentially expressed calmodulin genes

Multiple studies have shown that Ca^2+^ can increase the strength of the cell wall and maintain its integrity. Calcium signals generated by changes in intracellular Ca^2+^ concentration are mainly signal transduced by calcium target proteins. There are three main types of calcium target proteins in plants: calmodulin/calmodulin-like protein (CAM/CML), calcium-dependent protein kinase (CDPK), and calmodulin phosphatase B protein (CBL).

**Fig. 6:**
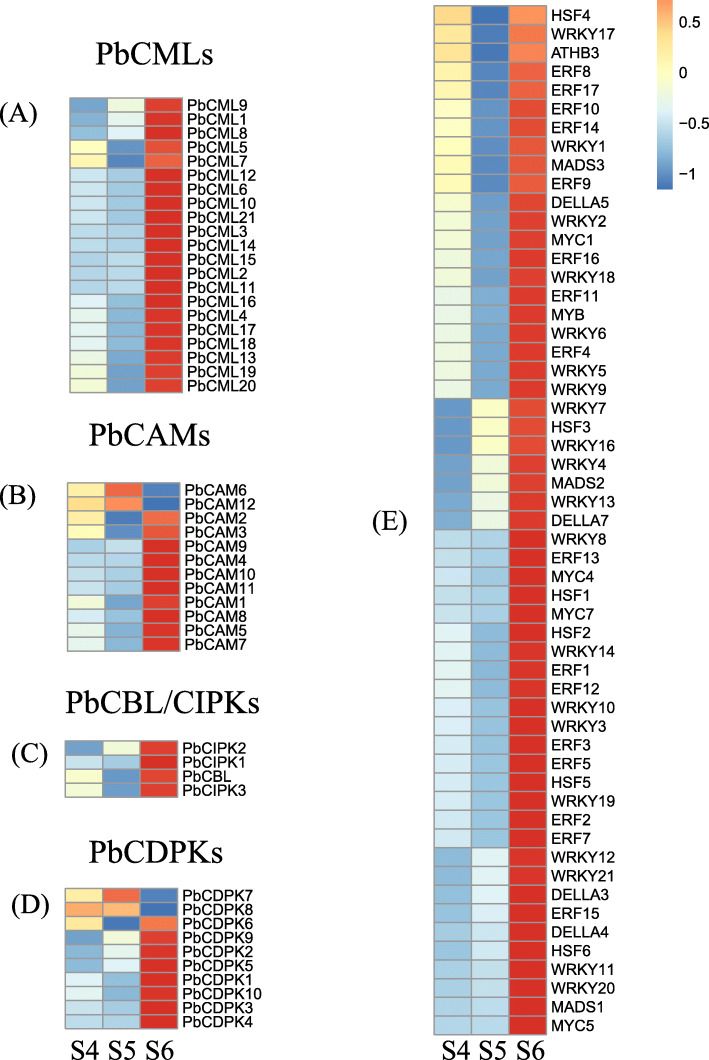
Heatmap of calcium-related differential expressed genes in transcriptome. The three columns represent the gene expression at S4, S5 and S6 respectively. (A) The expression pattern of CML gene family in callus among DEGs. (B) The expression pattern of CAM gene family in callus among DEGs. (C) The expression pattern of CBL/CIPK gene family in callus among DEGs. (D) The expression pattern of CDPK gene family in callus among DEGs. (E) The expression patterns of transcription factors in different genes in callus

In order to study the expression trend of calcium-related genes in pear callus after treatment, we used the software Pheatmap to cluster the selected calcium-related genes, and the results showed that the three main calcium target proteins were all involved in the response mode after treatment with calcium callus for 10 days (Fig. 6). The correspondence between the gene name and coding can be found in Table S2. Among them, the number of CMLs involved was the largest. Most CML genes were firstly none-regulated at the S5 level and then significantly up-regulated at the S6 level. The number of CAM DEGs and CDPK DEGs was similar, and most of them were significantly up-regulated at S6, while the number of CBL was only 1, which was also significantly up-regulated at S6 (Fig. 6-A, B, C, D). In addition, we found a large number of up-regulated transcription factors, such as WRKY, MYB, MYC, and DELLA, which are thought to be involved in the regulation of lignin synthesis (Fig. 6-E). This suggests that calcium increases the expression level of Ca^2+^ sensors, suggesting that calcium nitrate treatment does induce calcium signal transduction and promote secondary metabolic pathways as a whole. This is consistent with previous studies.

### Real-time quantitative PCR verification of DEGs

**Fig. 7:**
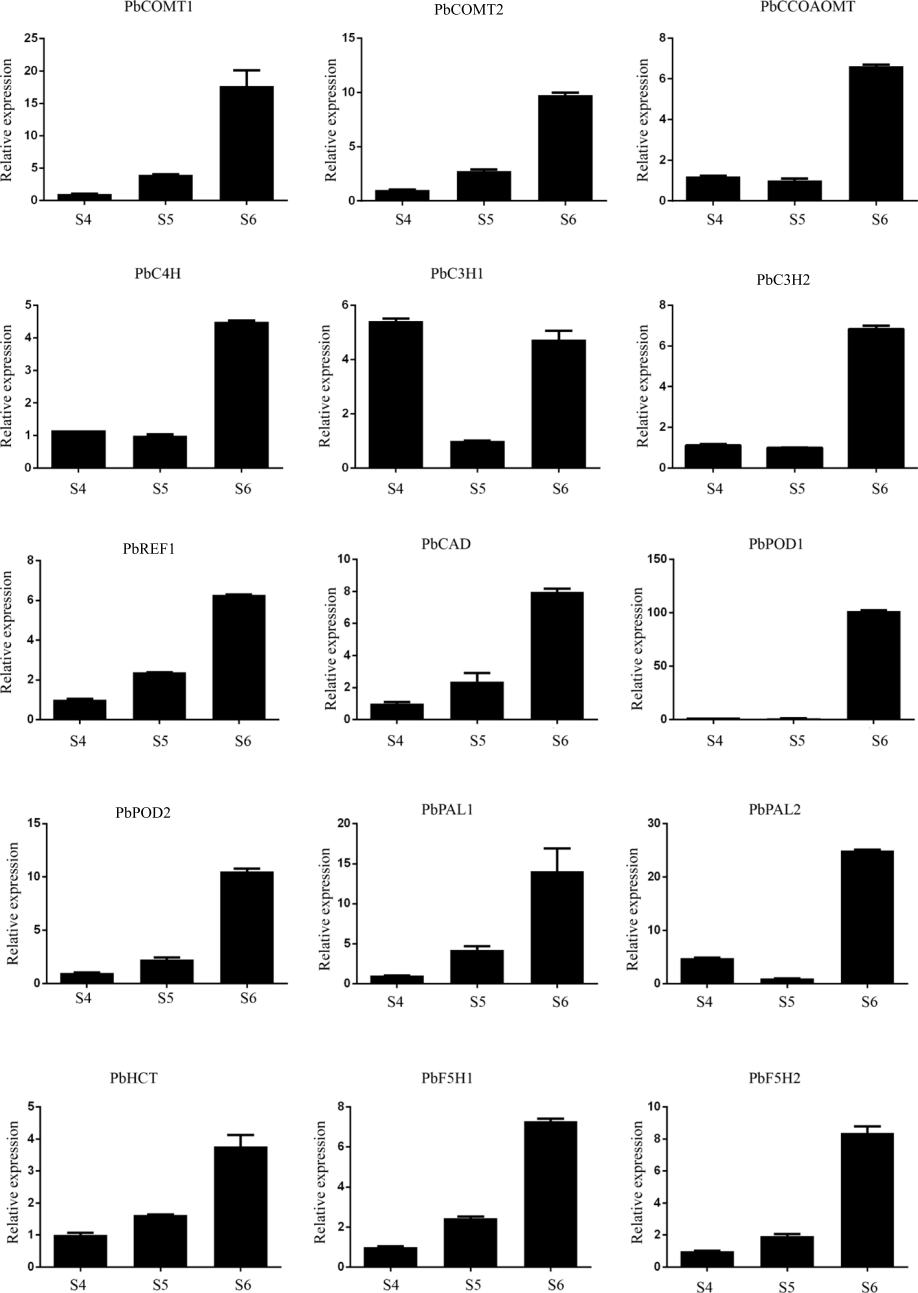
qRT-PCR analysis of DEGs in phenylpropanoid pathway

**Fig. 8:**
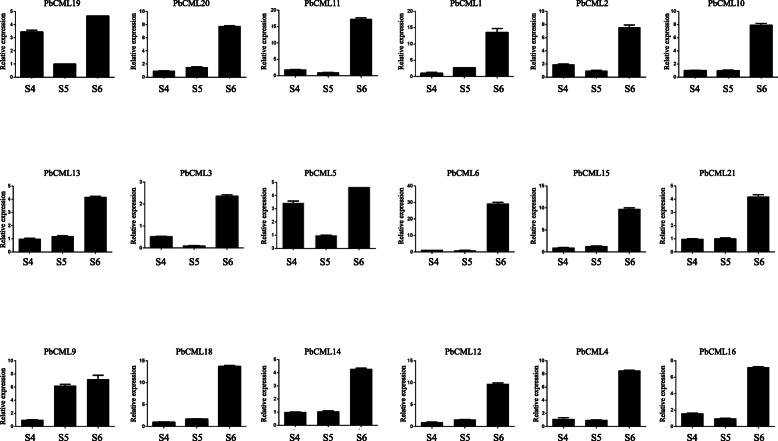
qRT-PCR analysis of DEGs in PbCMLs

In order to further verify the reliability of transcriptome sequencing expression profile data when treated with calcium nitrate, we selected 15 DEGs in the phenylpropanoid pathway and 18 *PbCMLs*, and verified the results of transcriptome sequencing by real-time fluorescence quantitative PCR. The results showed that the expression pattern of phenylpropanoid pathway DEGs was basically consistent with the transcriptome (Table S4). However, the PCR results of *PbC3H1* showed that the relative expression of S4 treatment was higher than transcriptome data (Fig. 7). The expression pattern of *PbCMLs* was consistent with the data in transcriptome. In addition, The relative expression of all *PbCMLs* were the highest in S6 (Fig. 8).

### Manipulating *PbCML3* expression increases stone cells in pear

**Fig. 9:**
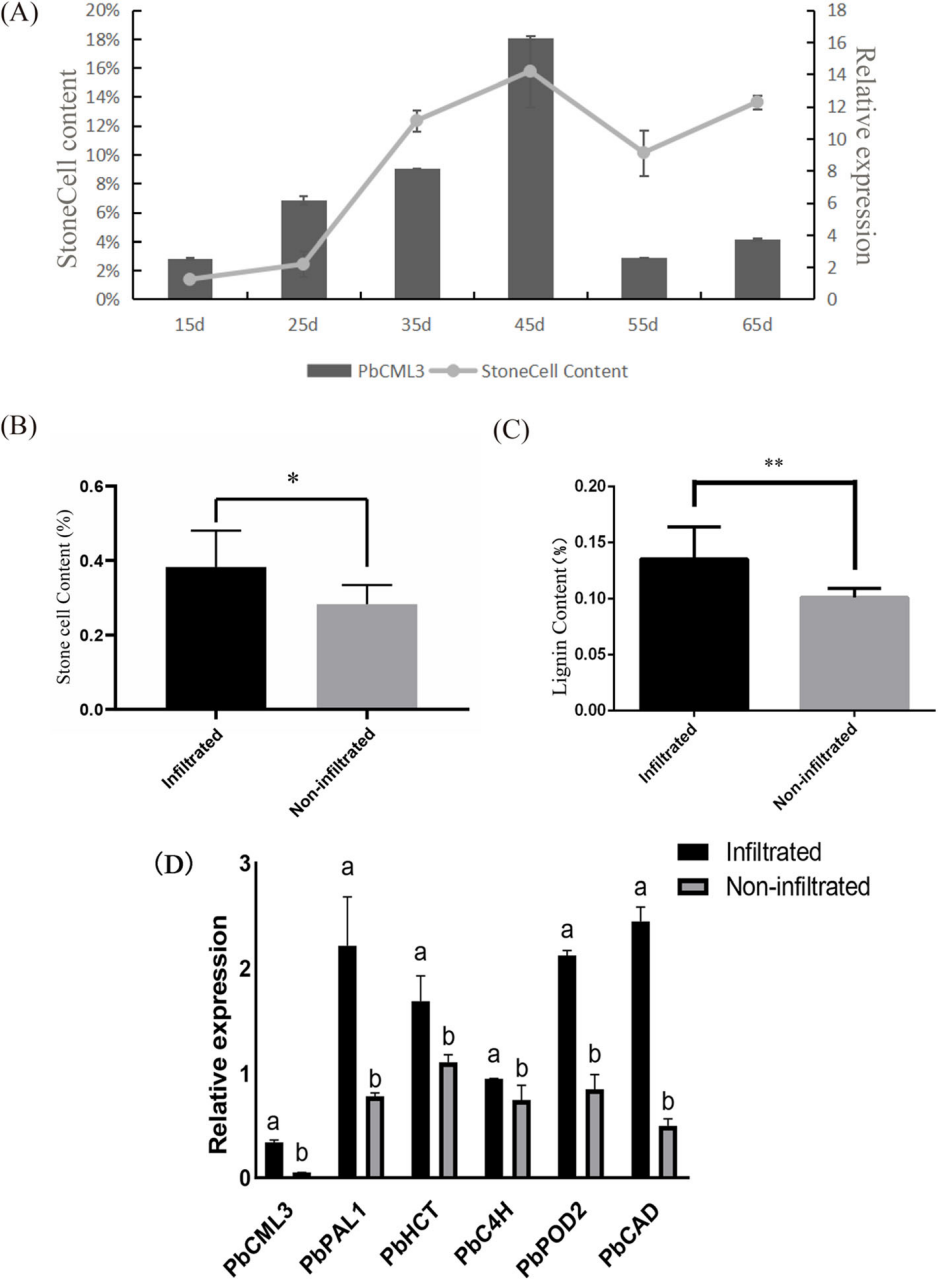
Association between pear stone cells and PbCML3. (**A**) Dynamic changes of stone cell content and PbCML3 in pear fruit development. (**B**) The content of stone cell in two sides. (**C**) The content of lignin in two sides. (**D**) Expression patterns of DEGs at injection site and non-injection site

‘Dangshansuli’ pear fruit stone cells begin to differentiate around 20 days after flowering, and peak at 40 to 50 days after flowering. The relative expression level of *PbCML3* in the fruit first increased and then decreased and reached its peak at 45 days after flowering, which was consistent with the expression rules of pear fruit stone cells (Fig. 9-A). Combining transcriptome data, it was further speculated that *PbCML3* had a certain regulatory effect on lignin and stone cell synthesis. *PbCML3* was overexpressed in pear fruits 35 days after blooming, one side of the fruit was injected with *35S-GFP-PbCML3* and the other side served as a control. After 10 days, the fruits were cut into slices and stained with phloroglucinol and hydrochloric acid (Fig. S2). The content of stone cells in the overexpressed *PbCML3* side was higher than that in the uninjected side (Fig. 9-B). By measuring the content of lignin in fruits in corresponding areas, it was found that the content of lignin in the overexpression vector region injected was higher than that in the region not injected, which increased by about 30.1%, reaching a significant difference. The relative expression of overexpression vector *PbCML3* in the fruit region was higher than that in the uninjected region, and increased by 574.1%. At the same time, lignin-related DEGs were up-regulated in the injection area (Fig. 9-D).

### PbCML3 localizes in plasma membrane

**Fig. 10:**
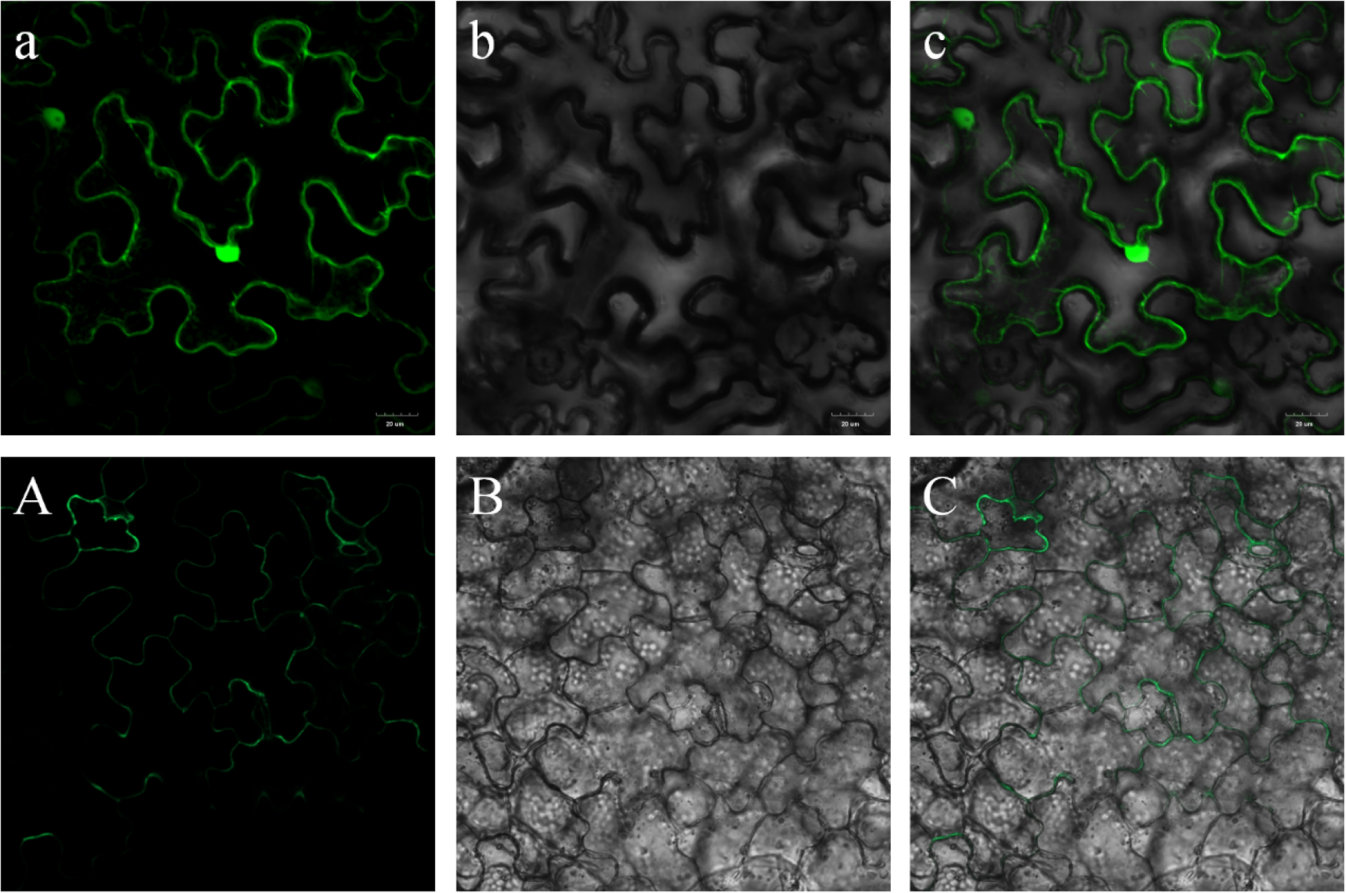
Subcellular localization of PbCML3 in tobacco mesophyll protoplasts. (**A**) Localization of 35S-GFP-PbCML3 fusion protein in tobacco mesophyll protoplasts. (**B**) FM 4–64 fluorescence (membrane marker) detected in the plasma membrane. (**C**) Merge of A and B

To clarify the function of the PbCML3 signal transporter, we analyzed the subcellular localization of PbCML3. The distribution of PbCML3 in the cell was determined by the method of instantaneous transformation of agrobacterium. After transient transformation into tobacco, 35S-GFP-PbCML3 fusion protein produced green fluorescence on the cell membrane, whereas the control displayed fluorescence in the cytosol and nuclei in the cells, indicating that PbCML3 was located on the cell membrane (Fig. 10).

## Discussion

### Exogenous 0.5% calcium nitrate promotes the process of secondary metabolism

Ca^2+^ has long been regarded as the second messenger of signal transduction [37], which plays an important physiological role in regulating plant cell elongation, cell membrane integrity, reactive oxygen metabolism [38]. In addition, it can affect plant photosynthesis, fruit quality, stem mechanical strength and stress resistance [11, 39, 40]. Application of 0.5% CaCl_2_ can inhibit the formation of stone cells [41]. However, different types of exogenous calcium may have different effects on lignin, treatment with 2% nano-CaCO_3_ was found to have effect on the mechanical strength of *P.lactiflora* inflorescence stems, with an observed increase of 30.73% [42]. In the present study, the treatment with 0.1% Ca (NO_3_)_2_ was found to have an influence on the lignin content of pear callus, but 0.5% Ca (NO_3_)_2_ was found to have an effect on the lignin content, with an observed increase on 10th day. Thus, the influence of lignin synthesis by exogenous calcium may have a potential relationship with the concentration on treatment. In the transcriptome, these 2889 DEGs were enriched into 16 KEGG metabolic pathways, including biosynthesis of secondary metabolites, phenylpropanoid biosynthesis, flavonoids biosynthesis, and signal transduction of plant hormones, etc. According to related research proof, phenylpropanoid metabolic pathway is one of the main path of lignin synthesis [5], all kinds of aromatic amino acids in this way will be formed by the chemical effect such as deamidation coumaric acid, coenzyme A, and then enter the flavonoids secondary metabolites biosynthesis pathway generated, thus forming lignin monomer and other secondary metabolites. The sequencing results were screened for genes related to phenylpropanoid pathway, most of which showed positive regulation. These results indicate that exogenous calcium nitrate can regulate phenylpropanoid pathway, and affect lignin synthesis. Moreover, As the upstream pathway of the phenylpropanoid for the synthesis of lignin, the shikimate pathway has also undergone a large number of gene upregulations. For example, arogenate dehydratase (ADT), regarded as a link between phenylalanine biosynthesis and lignin biosynthesis [43], is found in DEGs. Thus the regulation of exogenous calcium is not limited to phenylpropanoid pathway and lignin synthesis, but the entire secondary metabolism [44]. It had been demonstrated that exogenous calcium chloride treatment could increase the hardness of peony stems by enhancing lignin synthesis [9]. Also, the pear callus treated with calcium nitrate became brown and dull due to the more accumulation of lignin and other secondary metabolites.

### The regulatory network from calcium to stone cells in pear

**Fig. 11:**
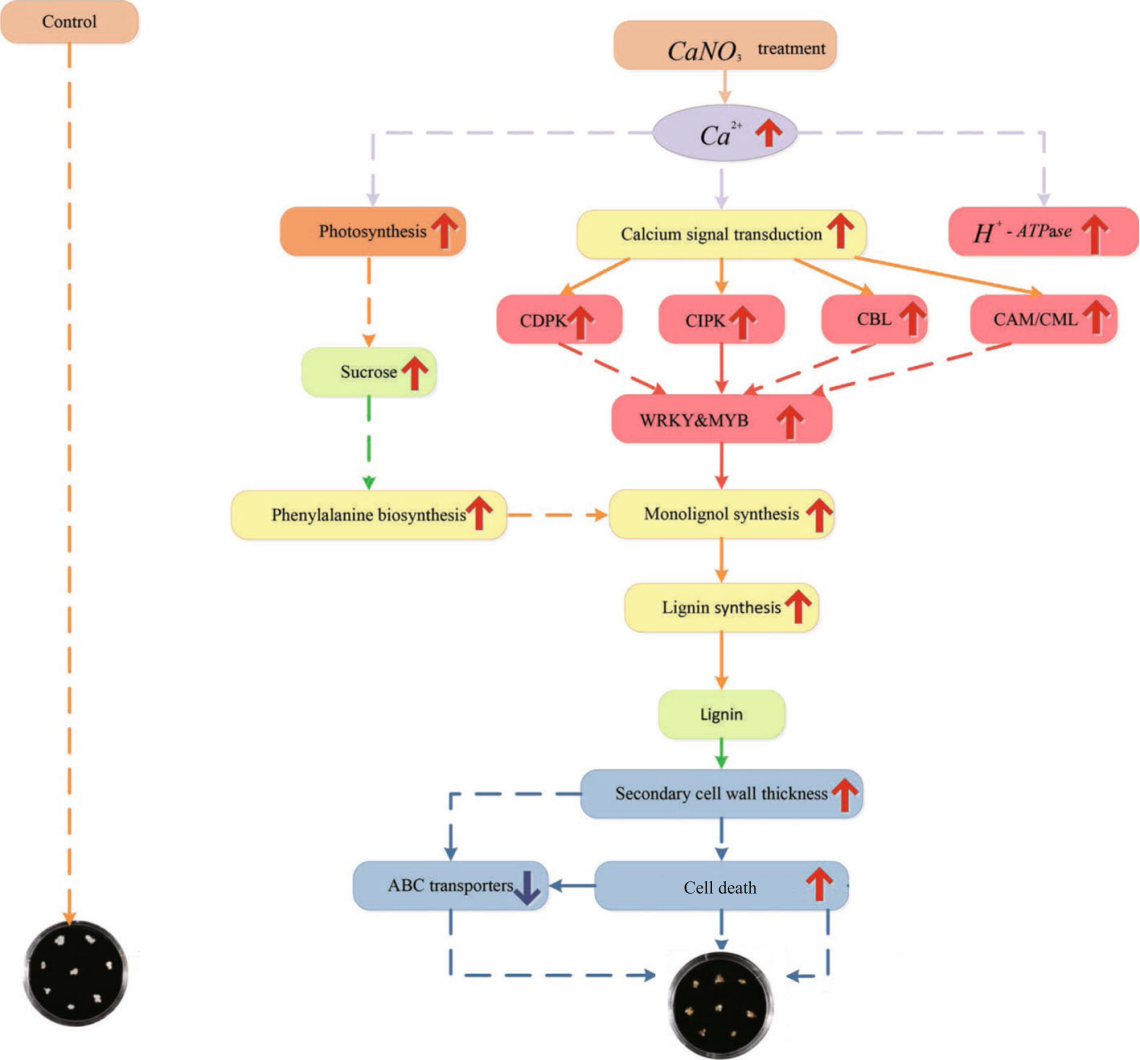
Proposed pathway for calcium-mediated enhancement of the lignin of pear callus

Lignin is the major component of the secondary cell walls and provides compression strength to the walls [3]. In pear, the accumulation of lignin is the key to the formation of stone cells [2]. In this study, we identified a lignin biosynthesis pathway that can enrich 9 DEGs (PAL, C4H, CAD, C3H, HCT, COMT, CCoAOMT, F5H and POD). Transcription factors regulating lignin synthesis, such as WRKY, MYB and DELLA, are also differentially expressed in this study. On previous studies, *WRKY36* and *WRKY102* are associated with repression of rice lignification [45], transcription factors *MYB58* and *MYB63* function in the control of lignin biosynthetic genes [46], the DELLA protein regulates the xylem fiber differentiation of *Arabidopsis thaliana* by binding to *KNAT1* [47], ERF transcription factor named *Ii049* positively regulates lignan biosynthesis in isatis indigotica through activating salicylic acid signaling and lignin pathway genes [48]. Moreover, RNA-seq identified five DEGs (CAM, CML, CIPK, CBL and CDPK) involved in calcium signal transduction, and they were up-regulated after 0.5% Ca (NO_3_)_2_ treatment. Furthermore, members of MYB families played roles as CAM-binding transcription factors [49]. Related research speculate that Ca^2+^ concentration increase can improve peony stem mechanical strength. Flower stem cell induced by calcium signal transduction, and it upregulate the NAC and MYB family, so as to promote secondary cell wall thickening [9]. Combined with our transcriptome data, this hypothesis is further proposed. Exogenous calcium regulates lignin by promoting calcium signal transduction and upregulating transcription factors, thus increasing lignin and stone cells in fruits. ABC transporters (ABCF), as potential monolignol transporter involved in lignin biosynthesis [50, 51], were found to be down-regulated in this processing and it may be caused by a certain degree of cell death during lignin synthesis. In the present study, a large number of secondary metabolic pathway genes and transcription factors of DEGs provide a network of exogenous calcium that causes metabolic pathway changes and leads to the accumulation of lignin in pear callus (Fig. 11).

### Overexpression of *PbCML3* promotes stone cells

The expression level of *PbCML3* at different developmental stages of pear fruit has demonstrated that *PbCML3* is expressed highest on 45th day (Fig. 9-A). Moreover, the expression pattern of *PbCML3* was consistent with the content of stone cells. It has been demonstrated that Lignin deposition is an important factor, which leads to the hardening of the cell wall of stone cells [2]. In our research, the stone cell and lignin content in *PbCML3* overexpression side was much higer than that of other side (Fig. 9-B, C). Thus, overexpression of *PbCML3* might cause changes in lignin pathway genes. Many previous reports had found that CML plays an important role in plant growth and development and stress, such as regulating the morphology and division of plant cells [26, 45] and responsing to salt, drought and low temperature stress [29]. *AtCML9* was found to promote plant innate immunity through flagellin-dependent signaling pathways [28]. The anti-stress function of plants is closely related to the synthesis of lignin [52]. The author hypothesized that *PbCML3* indirectly regulates lignin pathway genes. Furthermore, in the results of our qRT-PCR, the expression of important lignin synthesis DEGs (*PbPAL1, PbHCT, PbC4H, PbPOD2* and *PbCAD*) were found to upregulate on the *PbCML3* overexpressed side in fruit (Fig. 9-D). Those results prove that the CML gene can cause lignin accumulation during fruit development, which might be related to the regulation of stone cells content. Our research showed that *PbCML3* could promote lignin metabolism and thus stone cells accumulation. And then the specific regulation mechanism of CML on lignin content needs further study.

## Supplementary Information

**Additional file 1.** Table S1 Sequence of qRT-PCR primers. The “name” column of the table indicates the name of the gene in the manuscript, and the “Coding” column of the table indicates the serial number of the gene in pyrus genome

**Additional file 2.** Table S2 Gene numbers in heatmap. The serial numbers of all genes in the heatmap of Fig. 6. (Table S2a) Corresponding names of calcium-related genes. (Table S2b) Corresponding names of transcription factors

**Additional file 3.** Table S3 The number of transcriptome data uploaded to NCBI. For S1 ~ S6, each sample has three biological replicates, which are recorded with different SRR numbers. Transcriptome files are fastq files for paired sequencing

**Additional file 4.** Table S4 The expression matrix of transcriptome DEGs and raw data of qRT-PCR. (Table S4a) Transcriptome expression of related genes. The expression matrix of transcripts mentioned in the manuscript was provided, the unit of value is fpkm. (Table S4b) The original data of qRT-PCR. (Table S4c) Correlation between R-Seq and qRT-PCR in CMLs. The correlation of each CML DEGs were calculated with S4 ~ S6, and the values are all greater than 0.8

**Additional file 5.** Table S5 The fpkm matrix of transcriptome DEGs

**Additional file 6.** Table S6 The information of DEGs intersection. Table S6. The information of DEGs intersection. The DEGs intersection information with the two periods is provided. The table includes the KEGG function, chromosome position, positive and negative chain, gene length (bp), exon length (bp), CDS length (bp), amino acid Length (bp), number of exons, average length of exons (bp) and average length of introns (bp). The transcripts labeled “Novel” are not annotated by the genome

**Additional file 7.** Fig. S1 The Veen diagram of DEGs. The Veen diagram is drawn by Rscript, which shows the relationship set between the DEGs of S6/S4, S6S5, S3/S1 and S3/S2

**Additional file 8.** Fig. S2 Pear fruit injected with PbCML3. Each fruit is divided into injected side and non-injected side and the stone cells of the fruit are stained red

## Data Availability

The data of this study are available and the plant materials of this article have been frozen in the life sciences building of Nanjing Agricultural University. The transcriptome datasets supporting the conclusions of this article in the National Center for Biotechnology Information (https://www.ncbi.nlm.nih.gov/bioproject/PRJNA636000). Anyone who wants to obtain the public data in the research can contact us at taost@njau.edu.cn.

## Abbreviations

2,4-D: Auxin
AGAR: Agarose
ASP: Aspartate aminotransferase
C3H: P-coumarate 3-hydroxylase
C4H: Cinnamate-4-hydroxylase
CAD: Innamyl-alcohol dehydrogenase
CaCl_2_: Calcium chloride
CaCO_3_: Calcium carbonate
Ca (NO_3_)_2_: Calcium nitrate
CAM: Calmodulin protein
CBL: Calmodulin phosphatase B protein
CCOMT: Caffeoyl-CoA-3-O-methyl transferase
CCR: Cinnamoyl-CoA reductase
CDPK: Calcium-dependent protein kinase
CIPK: Calcineurin B-like interacting protein kinase
CML: Calmodulin-like protein
CSE: Caffeoy shikimate esterase
DAF: Days after full bloom
DAHPS: 3-deoxy-D-arabino-heptulosonate-7-phosphate-synthase
DEGs: Differentially expressed genes
F5H: Ferulate 5-hydroxylase
FPKM: Reads per kilobase of exon model per million mapped reads
GFP: Green fluorescent protein
GO: Gene ontology
HCT: Hydroxycinnamoyl transferase
KEGG: Kyoto encyclopedia of genes and genomes
KNA: Kanamycin
LAC: Laccase
LB: Luria-Bertani medium
NAA: 1-Naphthaleneacetic acid
NADPH: Nicotinamide adenine dinucleotide phosphate
NCBI: National Center of Biotechnology Information
PAL: Phenylalanine ammonia-lyase
PDT: Prephenate dehydratase
POD: Peroxidase
RPM: Revolutions per minute
qRT-PCR: Quantitative real-time polymerase chain reaction
SDH: Shikimate dehydrogenase
TDZ: Thidiazuron
RIP: Rifampicin
TRPS: Tryptophan--tRNA ligase
VC: Vitamin c
