# Supplementary material for: Transcriptome provides potential insights into how calcium affects the formation of stone cell in Pyrus
Source: BMC Genomics. 2021 Nov 17;22:831. doi: 10.1186/s12864-021-08161-5 (PMC8600858; doi:10.1186/s12864-021-08161-5)
Supplement: Supplementary file 7 — Additional file 7. Fig. S1 The Veen diagram of DEGs. The Veen diagram is drawn by Rscript, which shows the relationship set between the DEGs of S6/S4, S6S5, S3/S1 and S3/S2 [file 12864_2021_8161_MOESM7_ESM.pdf]

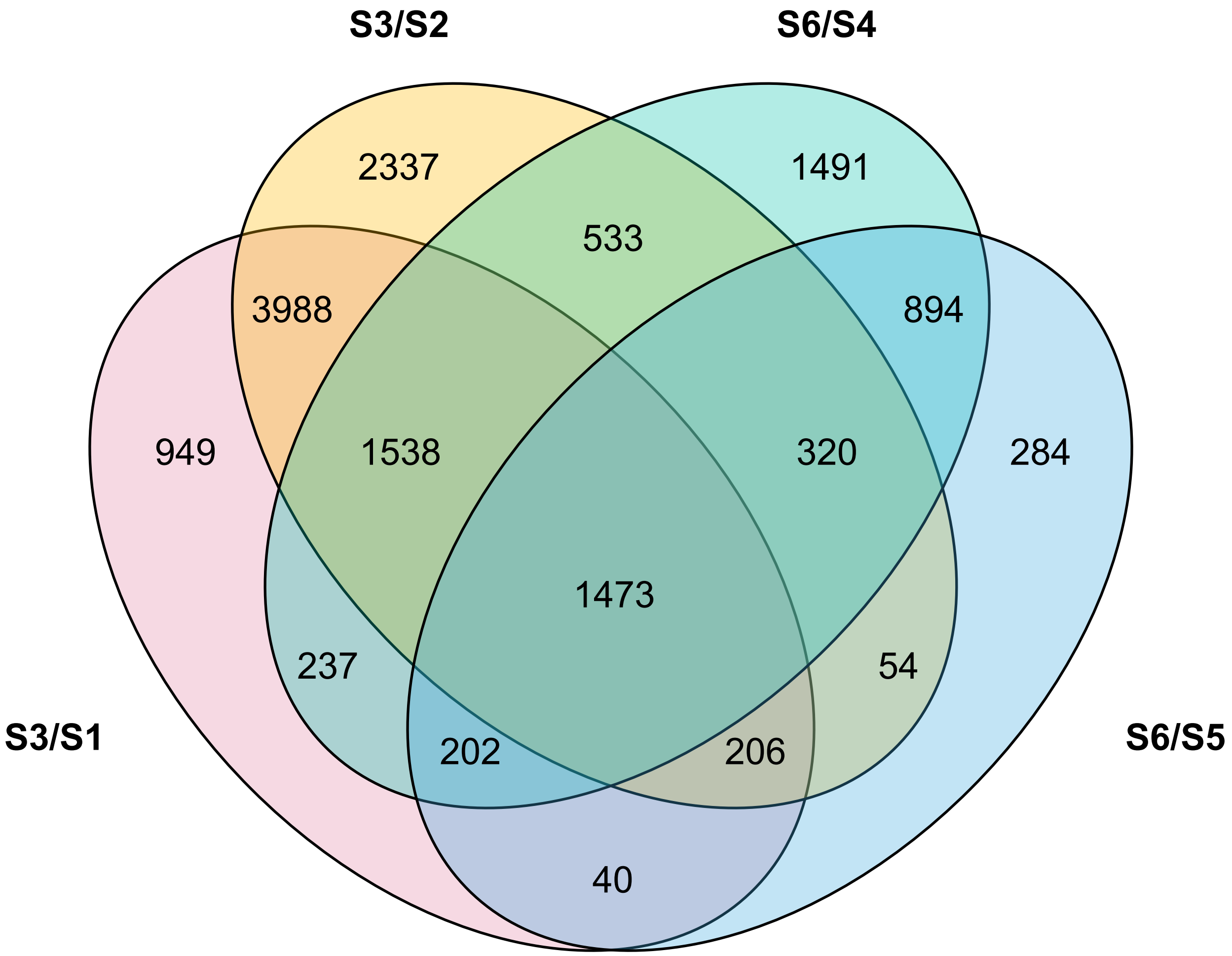

Figure S1. The Veen diagram of DEGs

The Veen diagram is drawn by Rscript, which shows the relationship set between the DEGs of S6/S4, S6S5, S3/S1 and S3/S2.
