# Supplementary material for: Transcriptome provides potential insights into how calcium affects the formation of stone cell in Pyrus
Source: BMC Genomics. 2021 Nov 17;22:831. doi: 10.1186/s12864-021-08161-5 (PMC8600858; doi:10.1186/s12864-021-08161-5)
Supplement: Supplementary file 8 — Additional file 8. Fig. S2 Pear fruit injected with PbCML3. Each fruit is divided into injected side and non-injected side and the stone cells of the fruit are stained red [file 12864_2021_8161_MOESM8_ESM.pdf]

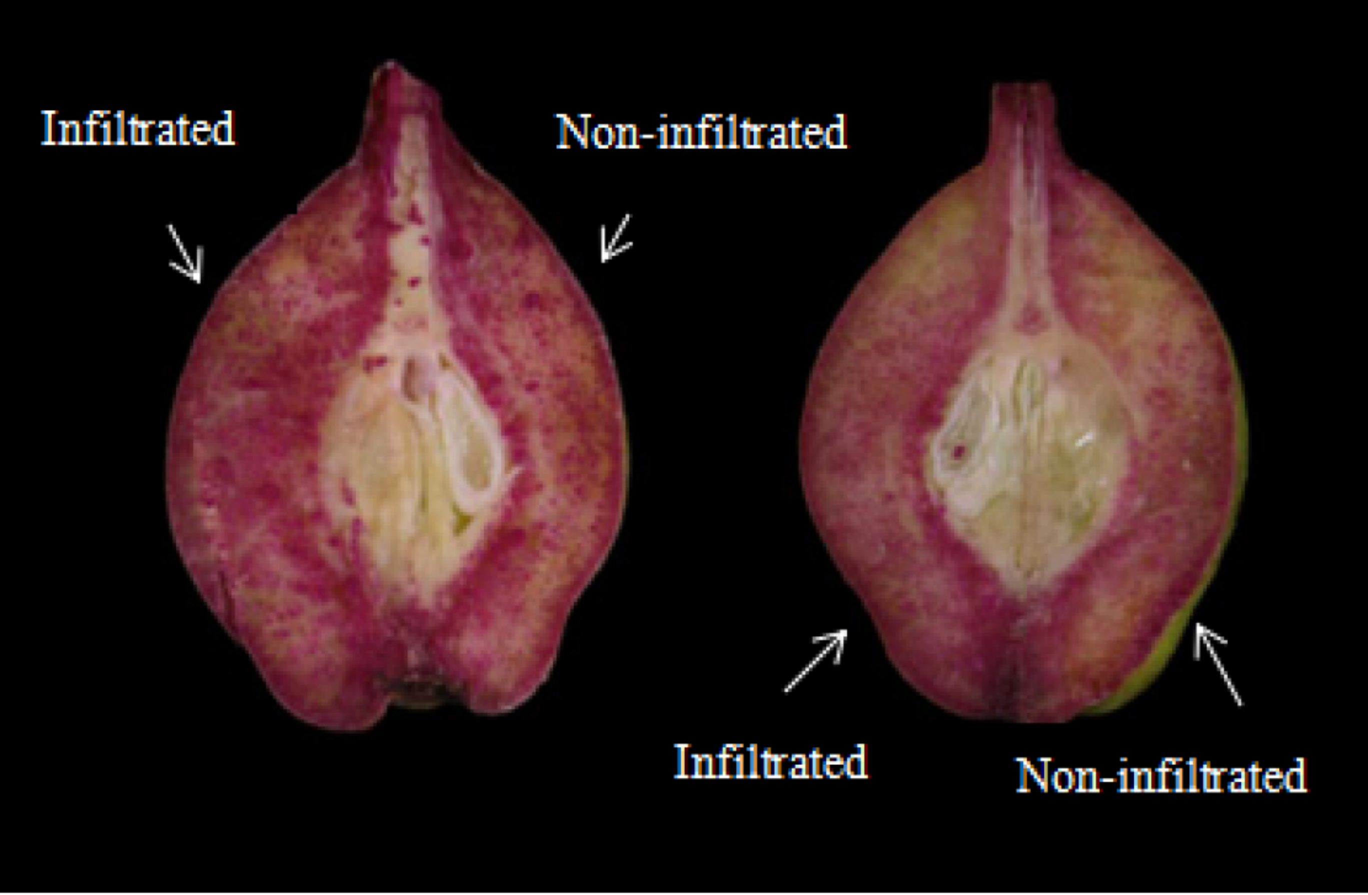

Figure S2. Pear fruit injected with PbCML3.

Each fruit is divided into injected side and non-injected side and the stone cells of the fruit are stained red.
